# Extensile lateral versus sinus tarsi approach for calcaneal fractures: A meta-analysis

**DOI:** 10.1097/MD.0000000000026717

**Published:** 2021-08-06

**Authors:** Chuangang Peng, Baoming Yuan, Wenlai Guo, Na Li, Heng Tian

**Affiliations:** a Department of Orthopedics, The Second Hospital of Jilin University, Changchun, Jilin, China; b Department of Hand Surgery, The Second Hospital of Jilin University, Changchun, Jilin, China.

**Keywords:** calcaneal fracture, extensile lateral approach (ELA), meta-analysis, outcomes, sinus tarsi approach (STA)

## Abstract

**Background:**

Calcaneal fractures are the most common tarsal bone fracture, and are often accompanied by heel pain, local swelling, tenderness, and inability to walk or stand. Surgical intervention results in better reconstruction of the calcaneal anatomy and reduces future complications; however, the optimal incision approach is still controversial. The incision is exposed better with extensile lateral approach (ELA), while the sinus tarsi approach (STA) causes fewer complications. The purpose of this meta-analysis is to compare the outcomes of STA and ELA.

**Materials and methods:**

Published trials comparing ELA and STA in calcaneal fractures were included in our analysis. The quality of each study was assessed using the revised Jadad scale and the Newcastle–Ottawa scale. Two researchers (CP and BY) independently extracted data from all selected studies. Fixed- or random-effects models with mean differences and odds ratios were used to pool the continuous and dichotomous variables to determine the heterogeneity of the included studies.

**Results:**

Calcaneal height and calcaneal width had high heterogeneity. Results showed that the incidence of incision complications in STA was lower than that in ELA (*P* < .001). There was high heterogeneity in operative time (*I*^2^ = 97%), length of hospital stay (*I*^2^ = 98%), Böhler angle (*I*^2^ = 80%), Gissane angle (*I*^2^ = 98%), and American Orthopaedic Foot & Ankle Society scores (*I*^2^ = 73%). No source of heterogeneity was found by sensitivity analysis, subgroup analysis, or regression analysis, and the random-effects model was used. STA operative time was significantly shorter than ELA (*P* < .001). Length of hospital stay after STA was significantly shorter than after ELA (*P* = .002). There was no statistical difference in the Böhler and Gissane angles between STA and ELA. Postoperative American Orthopaedic Foot & Ankle Society scores after STA were higher than after ELA (*P* = .01).

**Conclusions:**

Results show that, compared with ELA, STA is superior for treating calcaneal fractures due to anatomical reduction of the calcaneus, reduction of incision complications incidence, and shortened operative time and postoperative stay.

## 1 Introduction

Calcaneal fractures are the most common fractures of the tarsal bone, accounting for about 60% of all tarsal fractures.^[1]^ Most patients with calcaneal fractures land on their feet after falling from a height, which causes a vertical impact on the heel. Calcaneal fractures are often accompanied by heel pain, local swelling, tenderness, and the inability to walk and stand.^[2]^ Randomized controlled trials (RCTs)^[3,4]^ and meta-analyses^[5–7]^ have shown that surgical treatment is the preferred treatment for calcaneal fractures. And some new techniques are also applied to treat calcaneal fractures.^[8,9]^ Compared with non-surgical treatment, surgery results in better reconstruction of the anatomical structure of the calcaneus and reduces future complications.

Incision-related complications are an important limitation to the generalization of surgical treatment for calcaneal fractures. The traditional surgical approach consists of open reduction and internal fixation through the extensile lateral approach (ELA),^[10]^ where an L-shaped incision directly exposes the fracture site for repair.^[11]^ ELA can easily expose the incision, conducive to anatomical reduction. However, ELA can also damage the blood supply to the corners of the L-shaped flap, causing complications such as wound edge necrosis, nerve damage, and infection.^[12–14]^ To this end, clinical researchers have developed several small-incision, minimally invasive reduction techniques.^[15]^ The most common of these is the sinus tarsi approach (STA), which is performed through a small incision in the distal fibula that is anterior to the fibular tendon. While this approach mitigates the damage to the sural nerve and lateral calcaneal artery, some clinicians cite that inadequate exposure may affect the extent of reduction and, ultimately, overall functional recovery.^[16]^

There is increasing interest in this issue, and many meta-analyses have been conducted.^[17,9]^ However, we found errors in the literature inclusion^[18]^ that lead to doubts about the reliability of the conclusions presented from those studies. The present study aims to provide a more valid analysis by updating the literature and excluding inappropriate studies that have previously been used for meta-analysis. Herein, we performed a meta-analysis of STA versus ELA in terms of postoperative calcaneal height, postoperative calcaneus width, complications (marginal necrosis, wound infection, and nerve injury), operative time, postoperative hospital stay, postoperative Böhler angle, postoperative Gissane angle, the American Orthopaedic Foot & Ankle Society (AOFAS)-Ankle Hindfoot Scale score, and comprehensively evaluated the role of STA in the treatment of calcaneal fractures.

## 2 Methods

This meta-analysis was designed according to the Preferred Reporting Items for Systematic Reviews and Meta-Analyses guidelines ^[19]^ and was registered on the Prospero website (CRD: 42019122640). Analyses were based on previously published studies; thus, no ethical approval and patient consent are required.

### 2.1 Literature search strategies

Two authors (CP and BY) independently searched the online databases PubMed, Embase, and Cochrane on June 28, 2019 for studies comparing ELA and STA in the surgical treatment of calcaneal fractures using internal fixation. The keywords included: calcaneus, (fractures, bone), general surgery, surgical procedures, operative, and surgical wound. There were no restrictions on language, time, or any other parameters of the articles during literature retrieval. Meanwhile, the investigators also manually searched the references of relevant articles.

The details of the search strategy in PubMed are: Search ((((((((((((“Surgical Wound”[Mesh]) OR Surgical Wounds [Title/Abstract]) OR Wound, Surgical [Title/Abstract]) OR Wounds, Surgical [Title/Abstract]) OR Surgical Incision [Title/Abstract]) OR Incision, Surgical [Title/Abstract]) OR Incisions, Surgical [Title/Abstract]) OR Surgical Incisions [Title/Abstract]) OR approach [Title/Abstract])) AND ((((((((((((((“Surgical Procedures, Operative”[Mesh]) OR Operative Surgical Procedure [Title/Abstract]) OR Surgical Procedure, Operative[Title/Abstract]) OR Procedure, Operative Surgical[Title/Abstract]) OR Procedures, Operative Surgical[Title/Abstract]) OR Operative Procedures[Title/Abstract]) OR Operative Procedure[Title/Abstract]) OR Procedure, Operative[Title/Abstract]) OR Procedures, Operative[Title/Abstract]) OR Operative Surgical Procedures[Title/Abstract]) OR Surgery, Ghost[Title/Abstract]) OR Ghost Surgery[Title/Abstract])) OR (((“General Surgery”[Mesh]) OR Surgery, General[Title/Abstract]) OR Surgery[Title/Abstract]))) AND ((((((((((((((((“Fractures, Bone”[Mesh]) OR Broken Bones[Title/Abstract]) OR Bone, Broken[Title/Abstract]) OR Bones, Broken[Title/Abstract]) OR Broken Bone[Title/Abstract]) OR Bone Fractures[Title/Abstract]) OR Bone Fracture[Title/Abstract]) OR Fracture, Bone[Title/Abstract]) OR Spiral Fractures[Title/Abstract]) OR Fracture, Spiral[Title/Abstract]) OR Fractures, Spiral[Title/Abstract]) OR Spiral Fracture[Title/Abstract]) OR Torsion Fractures[Title/Abstract]) OR Fracture, Torsion[Title/Abstract]) OR Fractures, Torsion[Title/Abstract]) OR Torsion Fracture[Title/Abstract])) AND (((“Calcaneus”[Mesh]) OR Heel Bone[Title/Abstract]) OR Bone, Heel[Title/Abstract]).

### 2.2 Inclusion and exclusion criteria

Inclusion criteria consisted of 1) adult calcaneal fracture patients; 2) studies comparing postoperative functional outcomes of calcaneal fractures via ELA and STA; 3) studies reporting at least 1 of the following outcomes: postoperative calcaneal height, postoperative calcaneal width, complications (marginal necrosis, postoperative infection, and nerve injury), operative time, length of hospital stay, postoperative Böhler angle, postoperative Gissane angle, and AOFAS scores; and 4) cohort studies, controlled clinical trials, and RCTs.

Exclusion criteria consisted of 1) animal or cadaver studies; 2) studies in which valid data cannot be extracted or converted; 3) case reports; 4) systematic reviews and meta-analyses; and 5) conference papers without full text.

The 2 authors (CP and BY) independently screened titles and abstracts of the resulting studies based on inclusion criteria and excluded ineligible studies. Subsequently, the authors read the full texts independently to determine whether a study should be included in the final analysis. Any discrepancies that occurred were resolved through discussions with a third author until a consensus between all 3 authors (HT) was reached.

### 2.3 Outcome measures

The primary outcomes include calcaneal height, calcaneal width, and the complications (marginal necrosis, wound infection, and nerve injury), and the secondary outcomes include operative time, length of hospital stay, Böhler angle, and Gissane angle.

Calcaneal height and width are 2 important outcome indicators for predicting postoperative functional recovery. Calcaneal height is a radiographic parameter measured on the lateral radiographic view from the most posterior point of the tuberosity to the calcaneocuboid joint. There are 3 parts that should be calculated about the width of the calcaneus, including the width of the anterior calcaneus, middle calcaneus, and posterior calcaneus. The width of the calcaneus is defined as the horizontal line of each part on the same axial plane. And the loss of height and width of the calcaneus predicts postoperative dysfunctions, such as walking pain and varied load-bearing point of the heel.

Incision-related complications, operative time, and postoperative hospital stay are important considerations for the clinical application of STA. All these outcomes can be found after the surgery of calcaneal fractures, often used as indicators of recovery time.

The Böhler angle is an imaging index that serves as an anatomical landmark for the posterior articular surface of the subtalar joint. A decrease in the Böhler angle indicates a collapse of the load-bearing posterior articular surface of the calcaneus, which subsequently moves the center of gravity of the body forward and reduces the calcaneal height. The Gissane angle represents the angle between the anterior and posterior articular surfaces of the calcaneus. An increased Gissane angle indicates a collapse of the posterior surface of the calcaneus.

The AOFAS rating scale is used to score postoperative function; the maximum potential score is 100 points, and a score of 75 points or more is excellent.^[13,14,20–35]^

### 2.4 Data extraction

Two authors (CP and BY) independently extracted the data from each study that met the inclusion criteria. The following outcome measures were collected: postoperative calcaneal height, postoperative calcaneal width, complications (marginal necrosis, postoperative infection, and nerve injury), operative time, length of hospital stay, postoperative Böhler angle, postoperative Gissane angle, and AOFAS scores. Baseline data included: study time, type of study, mode of internal fixation, number of feet, the average age of patients, male to female ratio, and follow-up time. Discrepancies were resolved by discussions with a third author until an agreement was reached by all 3 authors (HT). We contacted the original authors via email to obtain the data that was not available in the original.

### 2.5 Quality assessment

Two authors (CP and BY) independently assessed the quality of the included literature using the modified Jadad scale for RCTs where scores 1 to 3 are considered low quality and scores 4 to 7 are high quality.^[36]^ Cohort studies were assessed using the Newcastle–Ottawa Scale,^[37]^ where scores 1 to 4 are considered low quality and scores 5 to 9 are high quality. Discrepancies were resolved by discussions with a third author (HT) until all 3 authors agreed.

### 2.6 Statistical analysis

Review Manager (Version 5.3) and Stata (Version 14) were used for data analysis. Dichotomous variables were assessed using risk ratios and 95% confidence interval (CI). Continuous variables were analyzed as the mean ± standard deviation and 95% CI. A value of *P* < .05 was considered statistically significant, and *I*^2^ values were calculated to assess heterogeneity across the studies. An *I*^2^ < 50% was considered low heterogeneity, and a fixed-effects model was used. An *I*^2^ > 50% indicated high heterogeneity, and the source of heterogeneity was determined through sensitivity analysis, subgroup analysis, and regression analysis. If the heterogeneity could not be reduced, a random-effects model was used. Sensitivity analysis was performed by removing the included studies one by one. Subgroup analysis was conducted based on different types of internal fixation, where there were 5 subgroups, including 1) ELA plate fixation vs STA screw fixation, 2) ELA plate fixation vs STA plate fixation, 3) ELA screw fixation vs STA plate fixation, 4) ELA mixed fixation vs STA screw fixation, and 5) ELA plate fixation vs STA mixed fixation. Egger test was used to check for publication bias if more than 10 articles were included in the data analysis.

## 3 Results

**Figure 1:**
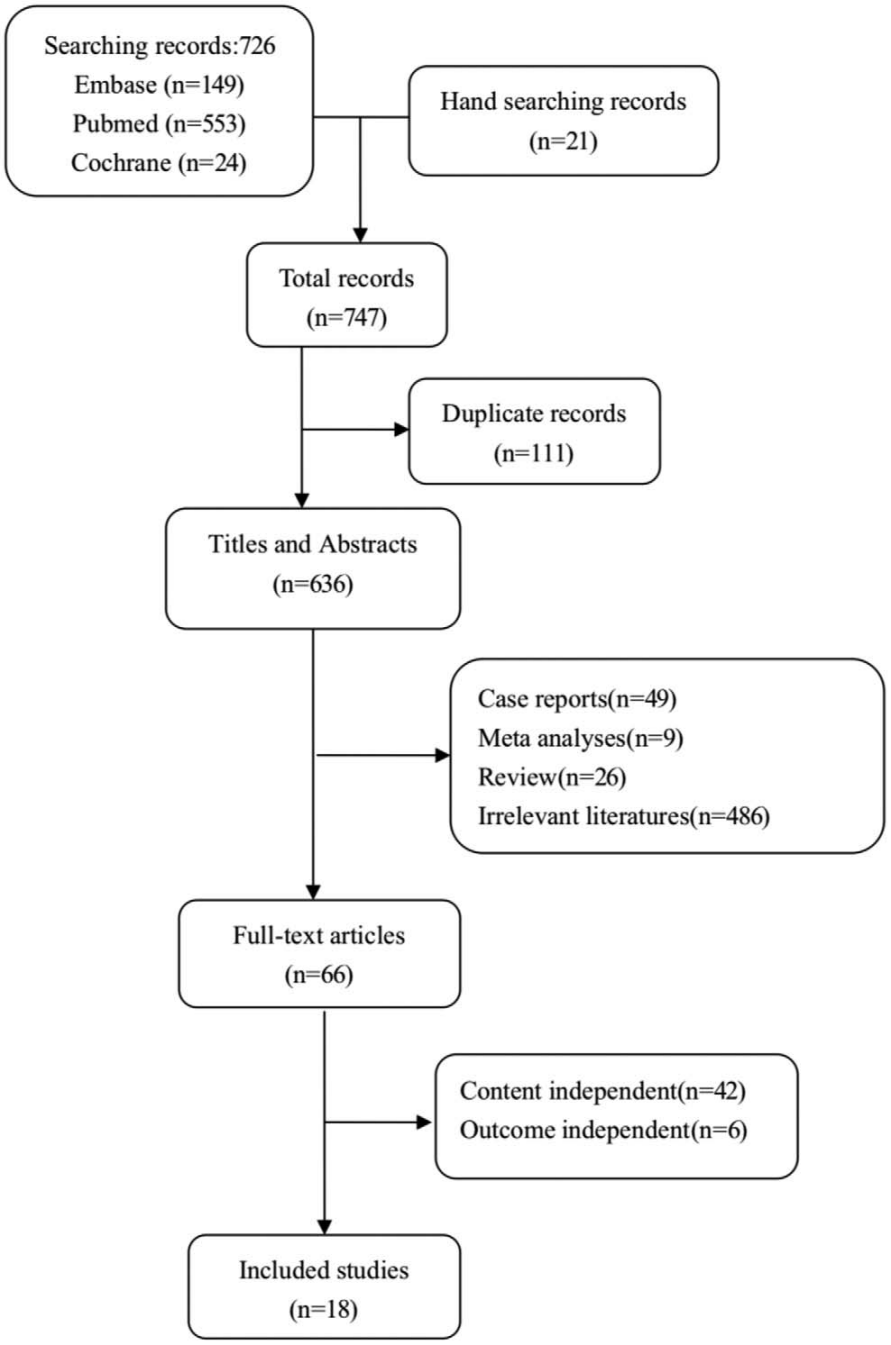
Literature selection. Flowchart for selection of included studies.

After an initial search, 726 articles were available from the following databases: 149 from Embase, 553 from PubMed, and 24 from Cochrane. We manually searched references of the 21 additional related articles. A total of 111 duplicate articles were removed, and another 570 articles were excluded by screening titles and abstracts. The remaining 66 articles were enrolled for full-text review, and an additional 48 articles (42 content-unrelated and 6 outcome-unrelated articles) were excluded thereafter. Thus, 18 articles were included in the final analysis (details of the literature selection are shown in Fig. 1).

Among the 18 included articles, 3 were written in Chinese, 1 was written in Korean, and the rest were written in English. Publication times ranged from August 2008 to February 2019. Across the 18 articles, 1648 calcaneus were reported: 838 for ELA and 810 for STA.^[13,14,20–35]^ The main characteristics and baseline data of the eligible studies are shown in Table 1.

**Table 1:** Main characteristics of all eligible studies included in the meta-analysis.

Studies	Period	Design	Fixed type (ELA/STA)	Fcet (ELA/STA)	Mean age (year) (ELA/STA)	Female (n) (ELA/STA)	Follow-up (month)	Quality score
AlexJ 2013^[13]^	2005.10–2008.12	CS	Plates; screws	79/33	42.2/46.4	12/7	50	5
Basile 2016^[20]^	2012.1–2012.12	RCT	Plates; plates	20/18	39.55/41.89	5/5	24	4
BinJia 2017^[21]^	2014.1–2016.12	RCT	Plates, plates	60.60	35.8/38.6	23/20	12	5
ChengL 2017^[22]^	2014.1–2015.1	RCT	Plates; plates	33/33	35.1/36.2	11/8		4
LiLH 2016^[24]^	2009.1–2014.1	RCT	Plates; plates	32/32	41/40	86	12	6
M.Weber 2008^[25]^	1995–2006	CS	Plates; screws (3 plates)	26/24	40,04/42.67		76	3′
Missa 2016^[26]^	2002–2012	CS	Plates; screw	23/27				7
Moon 2009^[14]^	2002.7–2007.2	CS	12 Plates and 8 screws; Screws	20/13	43.3/46.3	1/5	39.1	5′
Schepers 2017^[27]^	2012–2015	S	Screws; plates	6065	46	17/22	28	4
Sicm A 2017^[28]^	2012.1–2015.7	CS	Plates: plates	35/36	49/47	13/13	18	5
WuZp 2012^[29]^	2004.1–2009.12	CS	Plates; plates	170/213	41.49/39.42	E1/6	12	7
Xia 2014^[30]^	2007.1–2010.9	RCT	Plates; plates’	53/64	37/38	2/3	28	5
YangLei 2017^[31]^	2014.5–2016.5	CS	Plates; plates	50/54	36.835.9	14/16		8
Yeo 2015^[32]^	2004.9–2011.2	CS	Plates; screws	60:40	46	22/15	001	5′
ZShi 2013^[35]^		CS	Plates; plates	15/15	45.7/43.1	3/2	20	5
ZhouHC 2017^[33]^	2012.3–2015.2	CS	Plates; pcrews (2 plates)	37/28	43.8/43.6	9/7	15	5
ZhuHB 2013^[34]^	2011.3–2013.4	RCT	Plates; plates	18/20	36.4/36.6	7/5	15	3
Jinti Lin 2019^[23]^	2009.1–2016.4	CS	Plates; plates	47/35	38/36	7/7	50	7

Of the 6 RCTs, there was 1 low-quality study^[34]^ that did not describe the method of random number generation and 5 high-quality studies. Of the 12 cohort studies, 2 low-quality studies did not depict researcher choices clearly,^[25,27]^ and the other 10 were high-quality studies.

### 3.1 Calcaneal height

**Figure 2:**
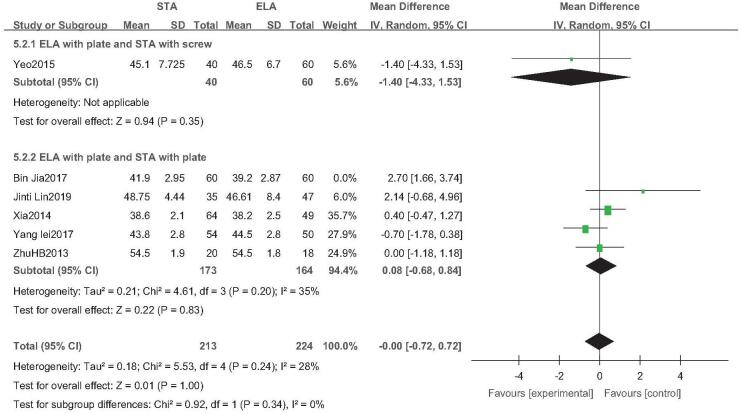
Calcaneal height analysis. Forest plot for the calcaneal height.

Six studies reported postoperative calcaneus height (STA: 273 feet; ELA: 284 feet).^[21,23,30–32,34]^ As *I*^2^ = 80%, indicating high heterogeneity, the study by Jia et al^[21]^ was removed for sensitivity analysis. *I*^2^ was reduced to 28%, and the random-effects model was used. Subgroup analysis revealed no statistical difference in the calcaneal height between STA and ELA, irrespective of the type of internal fixation used (odds ratio (OR) = 0.01; 95% CI: −0.72, 0.72; *P* > .05) (Fig. 2).

### 3.2 Calcaneal width

**Figure 3:**
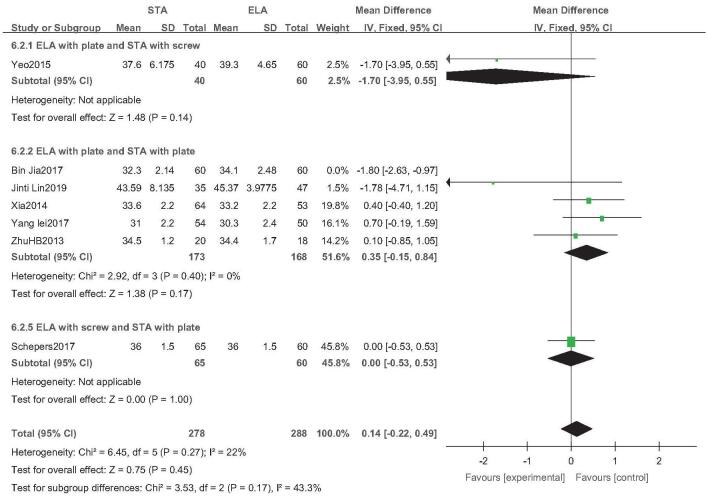
Calcaneal width analysis. Forest plot for the calcaneal width.

Seven studies reported postoperative calcaneal width (STA: 338 feet, ELA: 348 feet).^[19,21,25,28–30,32]^ As *I*^2^ = 75%, indicating high heterogeneity, sensitivity analysis was performed after removing Jia et al^[21]^*I*^2^ was then reduced to 22%, and the fixed-effects model was used. Subgroup analysis revealed no statistical difference in calcaneal width between STA and ELA, irrespective of the type of internal fixation used (OR = 0.14; 95% CI: −0.22, 0.49; *P* = .45) (Fig. 3).

### 3.3 Complications

**Figure 4:**
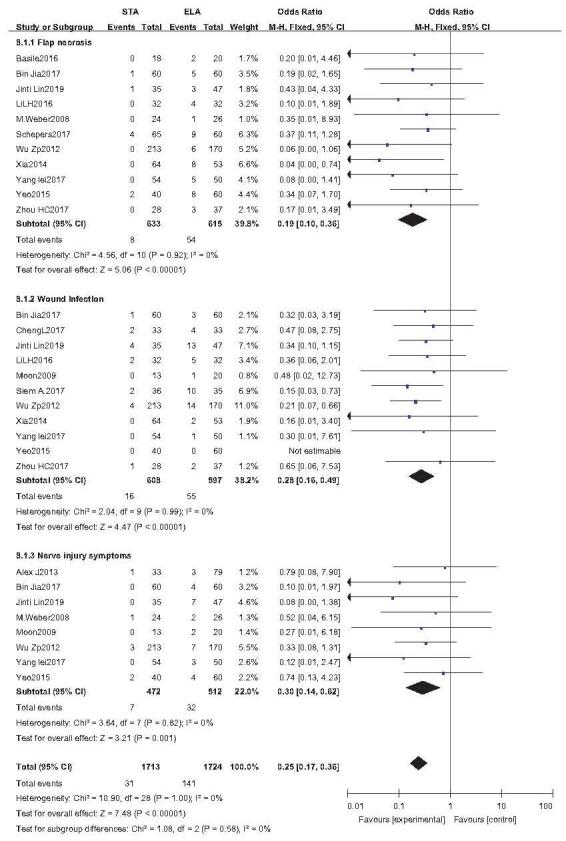
Postoperative complications analysis. Forest plot for postoperative complications.

Three common postoperative complications, including marginal necrosis, wound infection, and nerve injury, were analyzed by subgroup analysis. The results showed that the incidence of incision complications in STA was lower than that in ELA (OR = 0.25; 95%CI: 0.17, 0.36; *P* < .001) (Fig. 4).

### 3.4 Marginal necrosis

Eleven studies reported marginal necrosis (STA: 633 feet, ELA: 615 feet).^[20,21,23–25,27,29–33]^ As *I*^2^ = 0%, the fixed-effects model was used. The incidence of marginal necrosis in STA was significantly lower than that in ELA (OR = 0.19; 95% CI: 0.10, 0.36; *P* < .001).

### 3.5 Wound infection

Eleven studies reported wound infections (STA: 608 feet, ELA: 597 feet).^[14,21–24,28–33]^ As *I*^2^ = 0%, the fixed-effects model was used. The incidence of wound infection in STA was significantly lower than that in ELA (OR = 0.28; 95% CI: 0.16, 0.49; *P* < .001).

### 3.6 Nerve injury

Eight studies reported postoperative nerve injury (STA: 472 feet, ELA: 512 feet).^[13,14,21,23,25,29,31,32]^ As *I*^2^ = 0%, the fixed-effects model was used. The incidence of wound infection in STA was significantly lower than that in ELA (OR = 0.30; 95% CI: 0.14, 0.62; *P* < .001).

### 3.7 Operative time

**Figure 5:**
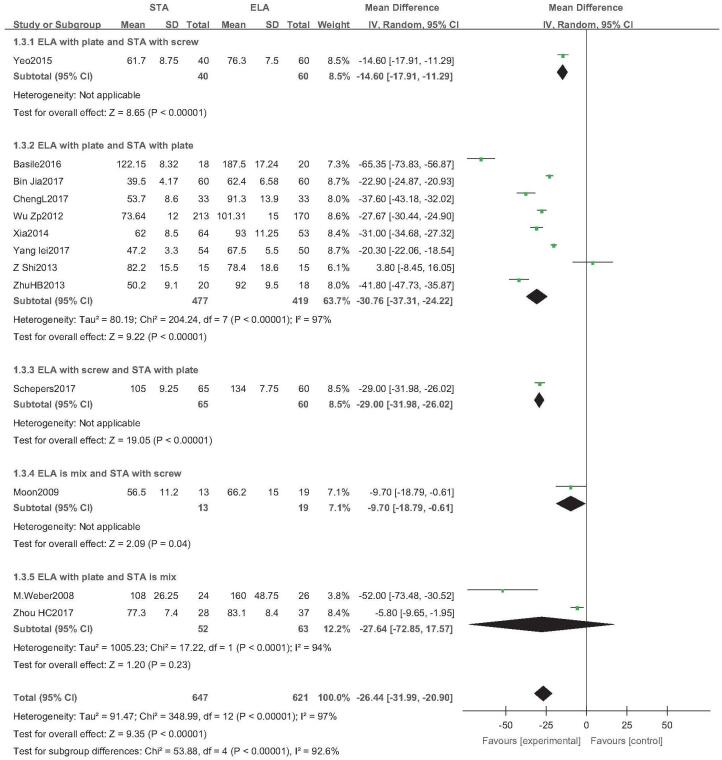
Operative time analysis. Forest plot for the operative time.

**Figure 6:**
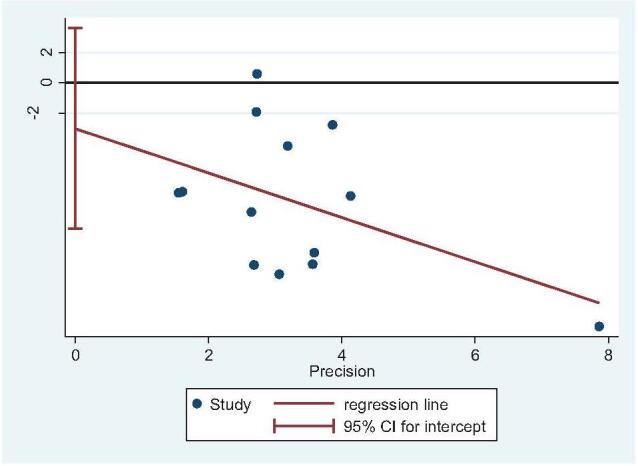
Publication bias analysis for operative time. Egger regression plot for operative time. (*P *< .05 indicated publication bias, *P* > .05 indicated no publication bias.)

Thirteen studies reported operative time (STA: 647 feet, ELA: 621 feet).^[14,20–22,25,27,29–35]^ There was high heterogeneity (*I*^2^ = 97%), but no source of heterogeneity was found by sensitivity analysis, subgroup analysis (Figure S1, Supplemental Digital Content), or regression analysis (Figure S2, Supplemental Digital Content), and the random-effects model was used. The operative time of STA was significantly lower than that of ELA (OR = −26.44; 95% CI: −31.99, −20.90; *P* < .001) (Fig. 5). Subgroup analysis (ELA plate fixation vs STA mixed fixation) revealed no difference in operative time between the 2 groups. An inconsistent fixation method used in the control group indicated poor stability in the outcomes. Egger test showed no publication bias (*P* = .337) (Fig. 6).

### 3.8 Length of hospital stay

**Figure 7:**
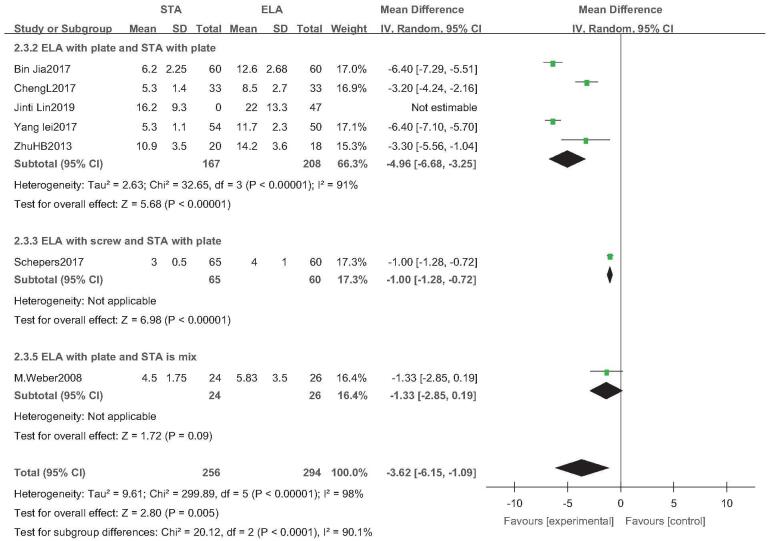
Length of hospital stay analysis. Forest plot for the hospital stay.

Seven studies reported length of hospital stay (STA: 291 feet, ELA: 294 feet).^[21–23,25,27,31,34]^ There was high heterogeneity (*I*^2^ = 98%), but no source of heterogeneity was found by sensitivity analysis, subgroup analysis (Figure S3, Supplemental Digital Content), and regression analysis (Figure S4, Supplemental Digital Content), and the random-effects model was used. Length of hospital stay in STA was significantly lower than that in ELA (OR = −3.83; 95% CI: −6.23, −1.42; *P* = .002) (Fig. 7). Subgroup analysis (ELA plate fixation vs STA mixed fixation) found no difference in the length of hospital stay between the 2 groups. There was poor stability in the outcomes as an inconsistent type of internal fixation was used in the control group. This may be caused by the high rates of complications after ELA surgery.

### 3.9 Böhler angle

**Figure 8:**
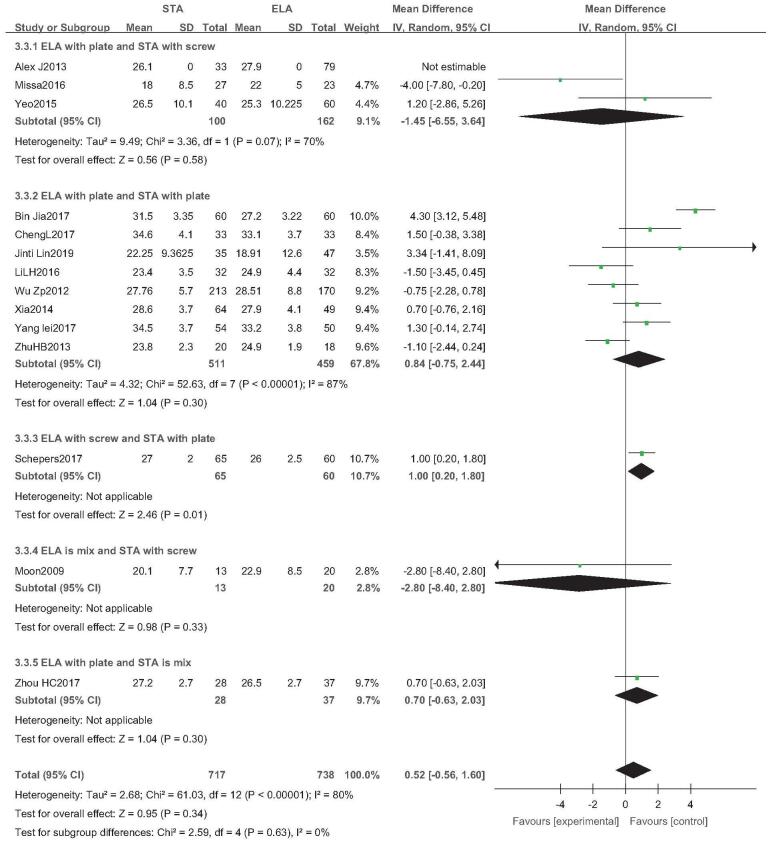
Böhler angle analysis. Forest plot for the Böhler angle.

**Figure 9:**
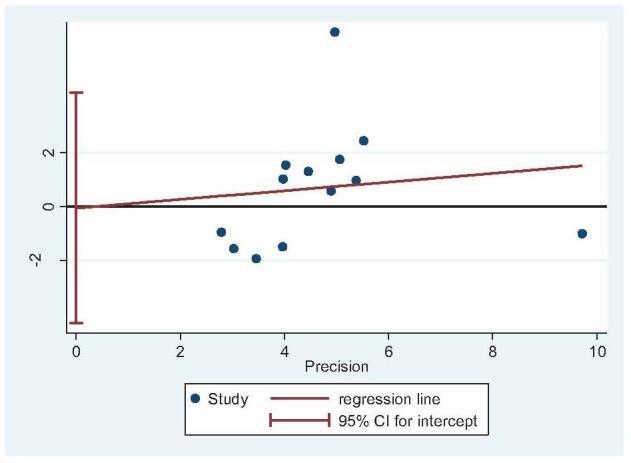
Publication bias analysis for Böhler angle. Egger regression plot for Böhler angle (*P* < .05 indicated publication bias, *P* > .05 indicated no publication bias.)

Fourteen studies reported the Böhler angle (STA: 717 feet, ELA: 738 feet).^[13,14,21–24,26,27,29–34]^ There was high heterogeneity (*I*^2^ = 80%), but no source of heterogeneity was found by sensitivity analysis, subgroup analysis (Figure S5, Supplemental Digital Content), and regression analysis (Figure S6, Supplemental Digital Content), and the random-effects model was then used. There was no statistical difference in the Böhler angle between the 2 groups (OR = 0.52; 95% CI: −0.56, 1.60; *P* = .34) (Fig. 8). Subgroup analysis (ELA screw fixation vs STA plate fixation) showed that the Böhler angle of ELA was slightly larger than that of STA, but it was only reported in 1 study, and further verification is required. Egger test showed no publication bias *(P* = .978) (Fig. 9).

### 3.10 Gissane angle

**Figure 10:**
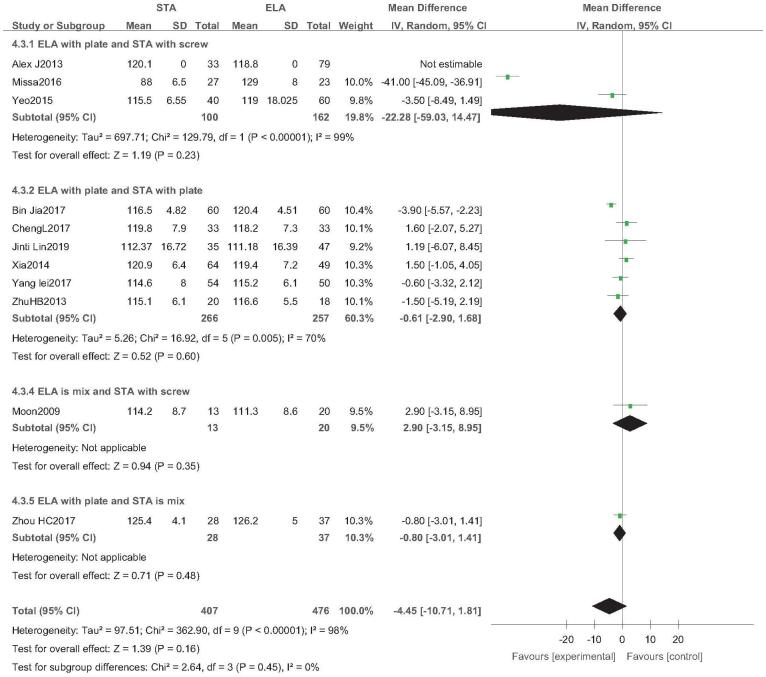
Gissane angle analysis. Forest plot for the Gissane angle.

**Figure 11:**
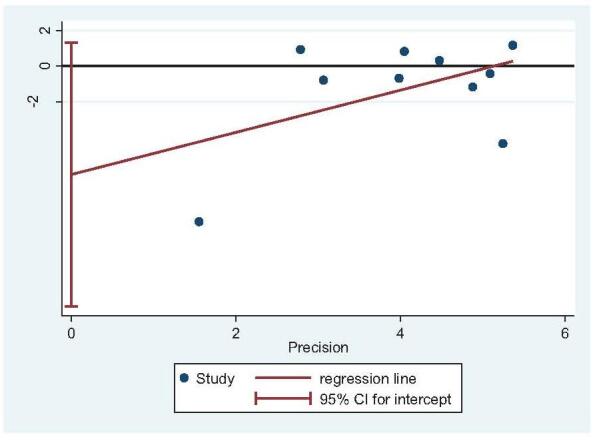
Publication bias analysis for Gissane angle. Egger regression plot for Gissane angle (*P* < .05 indicated publication bias, *P* > .05 indicated no publication bias.).

Eleven studies reported the Gissane angle (STA: 407 feet, ELA: 476 feet).^[13,14,21–23,26,30–34]^ There was high heterogeneity (*I*^2^ = 98%), but no source of heterogeneity was found by sensitivity analysis, subgroup analysis (Figure S7, Supplemental Digital Content), regression analysis (Figure S8, Supplemental Digital Content), and the random-effects model was used. Subgroup analysis showed that there was no significant difference in Gissane angle between STA and ELA, regardless of which type of internal fixation was used (OR = −4.45; 95% CI: −10.71, 1.81; *P* = .16) (Fig. 10). Egger test showed no publication bias (*P* = .095) (Fig. 11).

### 3.11 AOFAS score

**Figure 12:**
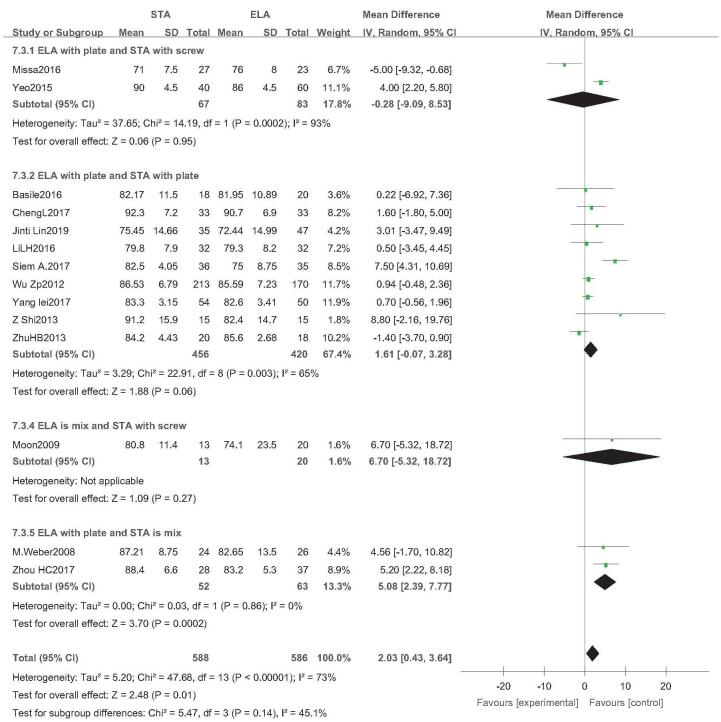
AOFAS Score analysis. Forest plot for the AOFAS score. AOFAS = American Orthopaedic Foot & Ankle Society.

**Figure 13:**
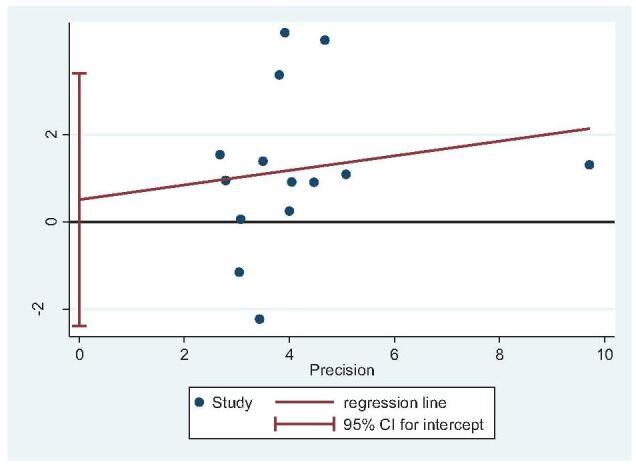
Publication bias analysis for AOFAS Score. Egger regression plot for the AOFAS score (*P* < .05 indicated publication bias, *P* > .05 indicated no publication bias.). AOFAS = American Orthopaedic Foot & Ankle Society.

Fourteen studies reported postoperative AOFAS scores (STA: 588 feet, ELA: 586 feet).^[14,20,22–26,28,29,31–35]^ There was high heterogeneity (*I*^2^ = 73%), but no source of heterogeneity was found by sensitivity analysis, subgroup analysis (Figure S9, Supplemental Digital Content), and regression analysis (Figure S10, Supplemental Digital Content). The random-effects model was then used. Postoperative AOFAS scores in STA were higher than those in ELA (OR = 2.03; 95% CI: 0.43, 3.64; *P* = .01) (Fig. 12). However, subgroup analysis (ELA plate fixation vs STA mixed fixation) showed higher AOFAS scores in STA than ELA; otherwise, all other subgroup analyses showed no statistical difference between the 2 groups. Egger test revealed no publication bias (*P* = .708) (Fig. 13).

## 4 Discussion

Our meta-analysis showed that surgical treatment utilizing an STA can effectively reduce incision-related complications, operative time, and hospital stay in patients with calcaneal fractures, which is similar to some other articles.^[18]^ However, this approach has similar therapeutic effects compared with ELA in terms of calcaneal height, calcaneal width, Gissane angle, Böhler angle, and AOFAS score.

A calcaneal fracture is the most common type of humeral fractures, and secondary diseases include traumatic arthritis, Achilles tendinitis, and tendonitis.^[2]^ Patients suffer from foot pain and movement disorders, and present with abnormal gait or claudication due to the abnormality in the whole force line of the foot, manifested as reduced foot arch and calcaneus valgus.^[2]^ Therefore, effective treatments are particularly important for calcaneal fracture patients. Conventional treatments include non-surgical and surgical options, and in recent years, surgical treatment for calcaneal fractures has become the preferred method. However, there is still a large dispute over the optimal surgical incisions and approaches. In the traditional ELA, an L-shaped incision of 8 to 10 cm is made to fully expose the fracture site, the subtalar joint, and bone tissue at the inner calcaneal wall, which is convenient for direct reduction and strengthening fixation of the fracture.^[38,39]^ In STA, a small incision of 3 to 4 cm is made parallel to the sole of the foot to expose the fracture, which is believed to better protect the blood supply and nerves around the incision. However, some researchers believe that, compared with ELA, an STA does not have obvious clinical advantages,^[40]^ especially in the recovery of important anatomical landmarks (calcaneal height, calcaneal width, Gissane angle, and Böhler angle).^[17,40,9]^ In addition, STA has less exposure to the fracture compared with ELA, which may result in decreased ability to ensure a proper anatomical reduction of the calcaneus and subtalar joints, and even influence long-term functional recovery.^[41,42]^ Even more compelling is the evidence that STA may increase wound complications.^[43]^ However, our meta-analysis showed that both surgical approaches were effective in reducing calcaneal fractures, and STA was not worse than ELA in restoring calcaneal height, calcaneal width, Gissane angle, and Böhler angle. Despite shortened operative times and hospital stays and reduced postoperative incision-related complications, STA had no obvious advantages in the long-term functional score at present. Further clinical trials are needed for verification.

In this study, primary outcome measures included calcaneal height, calcaneal width, and postoperative wound-related complications, including marginal necrosis, nerve injury, and wound infection. Compared with ELA, STA causes fewer wound complications due to the smaller surgical incision that avoids important vascular nerve structures and has less interference with bones and soft tissues around the fracture. The long incision used for ELA is more likely to damage the peroneal artery and its branches, resulting in insufficient blood supply to the lateral skin and a higher incidence of wound complications.^[43]^ We found high heterogeneity between the 2 groups in terms of calcaneal height and calcaneal width, However, sensitivity analysis showed that when excluding Jia et al,^[21]^ the heterogeneity was significantly reduced. There was no statistically significant difference between the 2 groups after the removal of heterogeneous sources.

In this study, there was shortened operative time with STA, which may be due to the fact that it is unnecessary to be overly careful when utilizing a small incision. In addition, the STA group had a shorter postoperative hospital stay when compared to the ELA group with longer incisions. In the latter group, much more soft tissue was damaged, accompanied by more severe incision complications, which subsequently resulted in a more complex recovery. The results of the meta-analysis showed no significant difference in Böhler and Gissane angles between STA and ELA. This indicates that although the incision in STA incision was smaller and operative time was shortened, STA did not affect the anatomical reduction of the calcaneus.

In this meta-analysis, AOFAS scores were used to assess postoperative functions, including pain, range of motion, walking distance, and stability. Previous studies have shown no significant difference in functional recovery between the 2 surgical approaches.^[17,9,25,26,29,40,44]^ ELA can fully expose the fracture site for a more comprehensive treatment of calcaneal injuries (especially subtalar joint injuries), at the expense of higher incision complication incidence and longer hospital stay. STA can mitigate some troubles in postoperative recovery, at the expense of a potential impact on the anatomical reduction of the calcaneus and subtalar joints due to limited surgical exposure. However, STA revealed no disadvantage in terms of anatomical reduction. Our meta-analysis data showed that the AOFAS score in the STA group was significantly higher than that in the ELA group. In other words, postoperative functional recovery was better after STA, consistent with the results of Zeng et al.^[18]^ However, our subgroup analysis showed no difference between ELA and STA in terms of ELA plate fixation vs STA screw fixation, ELA plate fixation vs STA plate fixation, and ELA mixed fixation vs STA screw fixation. Only ELA plate fixation vs STA mixed fixation showed the AOFAS score was higher in the STA group than the ELA group. However, this group contained only 2 studies and STA mixed fixation may result in poor stability of the experimental results, meaning that this result should be treated with caution.

### 4.1 Limitations

This meta-analysis only collected 6 RCTs (the rest being cohort studies), which may have biased the collected data. Additionally, the number of patients in some included studies is small,^[14,20,34,35]^ which may lead to unreliable results in the meta-analysis.

### 4.2 Strengths

In this meta-analysis, we established strict criteria for literature inclusion and exclusion, excluded inappropriate studies contained in previous meta-analyses, and only included RCT and cohort studies. During data analysis, we adopted various analytical methods such as sensitivity analysis, subgroup analysis, and regression analysis. In particular, we grouped the studies according to the types of research and internal fixation for subgroup analyses. This study is currently the most comprehensive meta-analysis concerning the outcomes of calcaneal fractures.

## 5 Conclusions

Our meta-analysis results show that, compared with ELA, an STA is superior in the treatment of calcaneal fractures, due to effective anatomical reduction of the calcaneus, effective reduction of the incidence of incision complications, and shortened operative time and postoperative hospital stay.

## Author contributions

**Conceptualization:** Chuangang Peng.

**Data curation:** Chuangang Peng, Baoming Yuan.

**Formal analysis:** Chuangang Peng, Wenlai Guo, Heng Tian.

**Investigation:** Chuangang Peng, Wenlai Guo, Heng Tian.

**Methodology:** Baoming Yuan, Wenlai Guo.

**Project administration:** Baoming Yuan.

**Resources:** Baoming Yuan, Na Li, Heng Tian.

**Software:** Baoming Yuan, Wenlai Guo, Na Li, Heng Tian.

**Supervision:** Wenlai Guo, Heng Tian.

**Writing – original draft:** Na Li.

**Writing – review & editing:** Na Li.

## Supplementary Material

Supplemental Digital Content
