# Supplementary material for: Extensile lateral versus sinus tarsi approach for calcaneal fractures: A meta-analysis
Source: Medicine (Baltimore). 2021 Aug 6;100(31):e26717. doi: 10.1097/MD.0000000000026717 (PMC8341246; doi:10.1097/MD.0000000000026717)
Supplement: Supplemental Digital Content [file medi-100-e26717-s001.doc]

251658240

Supplemental Digital Content (S1 Figure)

251658240


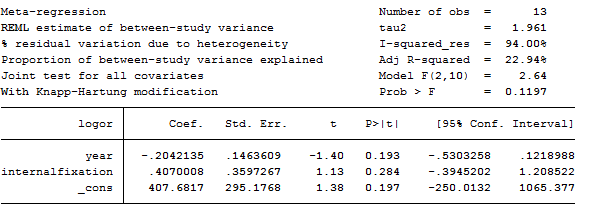


Supplemental Digital Content (S2 Figure)

251658240

Supplemental Digital Content (S3 Figure)

251658240


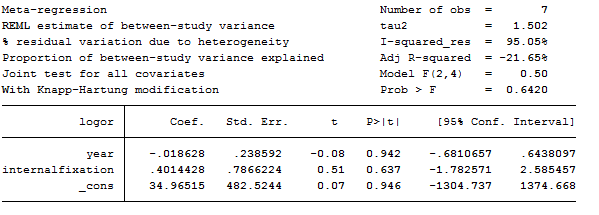


Supplemental Digital Content (S4 Figure)

251658240

Supplemental Digital Content (S5 Figure)

251658240


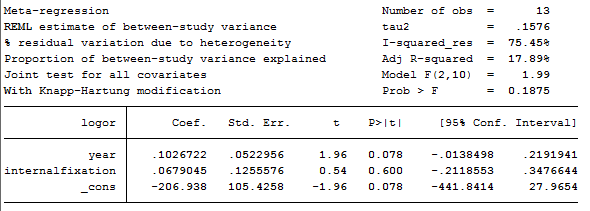


Supplemental Digital Content (S6 Figure)

251658240

Supplemental Digital Content (S7 Figure)

251658240


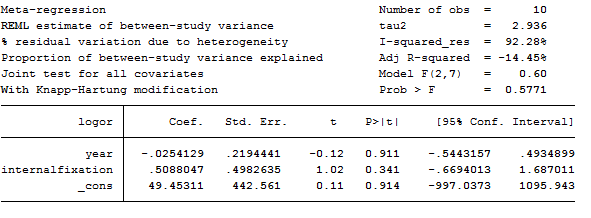


Supplemental Digital Content (S8 Figure)

251658240

Supplemental Digital Content (S9 Figure)

251658240


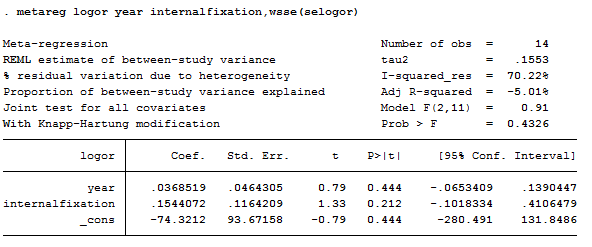


Supplemental Digital Content (S Figure)
